# Developing and testing a robotic MRI/CT fusion biopsy technique using a purpose-built interventional phantom

**DOI:** 10.1186/s41747-022-00308-7

**Published:** 2022-11-22

**Authors:** Edward W. Johnston, Nicos Fotiadis, Craig Cummings, Jodie Basso, Toby Tyne, Joost Lameijer, Christina Messiou, Dow-Mu Koh, Jessica M. Winfield

**Affiliations:** 1 Royal Marsden Hospital, 203 Fulham Road, London, SW3 6JJ UK; 2 Institute of Cancer Research, 123 Old Brompton Road, London, SW73RP UK

**Keywords:** Image-guided biopsy, Magnetic resonance imaging, Multimodal imaging, Phantoms (imaging), Robotics

## Abstract

**Background:**

Magnetic resonance imaging **(**MRI) can be used to target tumour components in biopsy procedures, while the ability to precisely correlate histology and MRI signal is crucial for imaging biomarker validation. Robotic MRI/computed tomography (CT) fusion biopsy offers the potential for this without in-gantry biopsy, although requires development.

**Methods:**

Test–retest T1 and T2 relaxation times, attenuation (Hounsfield units, HU), and biopsy core quality were prospectively assessed (January–December 2021) in a range of gelatin, agar, and mixed gelatin/agar solutions of differing concentrations on days 1 and 8 after manufacture. Suitable materials were chosen, and four biopsy phantoms were constructed with twelve spherical 1–3-cm diameter targets visible on MRI, but not on CT. A technical pipeline was developed, and intraoperator and interoperator reliability was tested in four operators performing a total of 96 biopsies. Statistical analysis included T1, T2, and HU repeatability using Bland–Altman analysis, Dice similarity coefficient (DSC), and intraoperator and interoperator reliability.

**Results:**

T1, T2, and HU repeatability had 95% limits-of-agreement of 8.3%, 3.4%, and 17.9%, respectively. The phantom was highly reproducible, with DSC of 0.93 *versus* 0.92 for scanning the same or two different phantoms, respectively. Hit rate was 100% (96/96 targets), and all operators performed robotic biopsies using a single volumetric acquisition. The fastest procedure time was 32 min for all 12 targets.

**Conclusions:**

A reproducible biopsy phantom was developed, validated, and used to test robotic MRI/CT-fusion biopsy. The technique was highly accurate, reliable, and achievable in clinically acceptable timescales meaning it is suitable for clinical application.

**Supplementary Information:**

The online version contains supplementary material available at 10.1186/s41747-022-00308-7.

## Key points

- The biopsy phantom had magnetic resonance imaging visible/computed tomography invisible targets and was highly reproducible.
- Four operators with different levels of experience sampled all targets (96/96) successfully.
- The fastest operator biopsied all twelve targets within 32 min.

## Background

Magnetic resonance imaging (MRI) confers exquisite soft-tissue contrast and the ability to probe multiple biophysical characteristics in a single examination [1] and may direct biopsy sampling of deterministic tumour regions. To understand the underpinnings of MRI signals, imaging biomarker studies [2] and radiogenomic studies [3] also require precisely localised pathological correlation with a view toward ‘digital biopsy’ [4, 5]. Whilst in-gantry biopsy could achieve these aims, biopsies are usually performed under ultrasound or computed tomography (CT) guidance due to lower cost, wider availability, and ease of use [6].

MRI/CT-fusion provides a potential solution, exploiting the advantages of both imaging modalities, although remains largely undeveloped. A CT-guided interventional robot (MAXIO, Perfint, Chennai, IN) has capabilities in multiplanar planning, stereotactic targeting, and new work-in-progress MRI/CT-fusion software, which is still in development outside of United States Food and Drug Administration labelled use and has not yet been used clinically. The IDEAL recommendations for new interventional procedures state that preclinical research should be conducted prior to first-in-human studies [7] and a standardised interventional phantom would be a suitable means of achieving this, assisting with the clinical development and validation of the fusion software. However, commercially available phantoms cannot be biopsied, degrade with use and have components which can be seen on both MRI and CT, making image fusion unnecessary [8].

Here, we design and validate a purpose-built MRI/CT fusion biopsy phantom, containing targets that can be seen on MRI but not on CT, can be biopsied and assessed for diagnostic material. We then use the phantom to establish a biopsy technique pipeline, test procedural reliability, and minimise risk prior to potential clinical translation.

## Methods

This prospective study was carried out at the Royal Marsden Hospital between January and December 2021. Ethical review was waived due to non-clinical nature. Data generated or analysed during the study are available from the corresponding author by request.

### Phantom development

#### Material investigation (tray experiments)

**Fig. 1:**
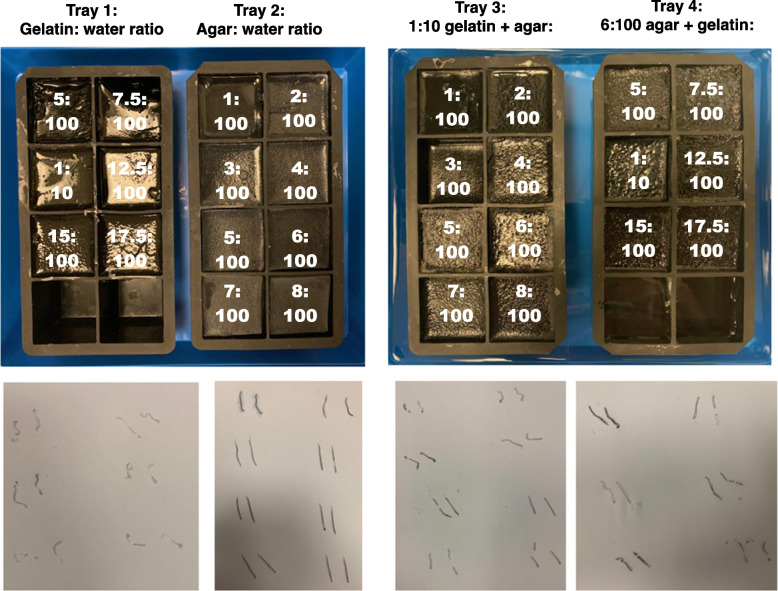
Concentrations and core adequacy of gelatin, agar, and mixed gelatin/agar samples. Concentrations (shown as white text overlaid on images) denote ratios of gelatin or agar to water, by weight ratio. Mixed gelatin/agar samples contain equal volumes of gelatin and agar solutions at specified concentrations. Biopsy samples taken on day 1 are shown below each tray

High-strength (250 ‘bloom’) bovine gelatin and agar were chosen as potential materials due to non-toxicity, low cost, availability, prior use in interventional phantoms, and the ability to generate different MRI contrast [9, 10]. We investigated a range of concentrations, including mixed solutions, in silicone trays (100 mL per cube) as controlled experiments (Fig. 1). Trays underwent both CT and MRI to determine their attenuation and relaxation times respectively, and biopsy for core quality (Fig. 1).

All CT acquisitions in this study were carried out using the same protocol. Images were acquired in the axial plane, using a CT scanner with 64 detector rows (Definition Edge, Siemens Healthineers, Erlangen, Germany) and a 3-mm slice thickness with 1-mm slice interval.

Room temperature trays were placed in a bath of doped water (770 mg × L^−1^ CuSO_4_, 2,000 mg × L^−1^NaCl) and magnetic resonance images were acquired using a 1.5-T scanner (Magnetom Aera, Siemens Healthineers, Erlangen, Germany). T1 relaxation times were estimated using inversion-prepared turbo spin-echo (TSE) sequences (inversion times 35−3,000 ms, repetition time 4,500 ms) and T2 relaxation times using a multi-contrast spin-echo sequence (echo times 10−320 ms, repetition time 5,000 ms) as described previously [11]. Trays were imaged twice on day 1 after manufacture (2 h apart) to assess repeatability, and day 8 to assess medium-term stability over a typical period of use.

T1 and T2 relaxation times were estimated using in-house software (Adept, ICR). Curves were fitted pixel-by-pixel and median values of a region-of-interest (750 mm^2^) calculated for each cube. Hounsfield units (HU) were measured at the same position using CT images, acquired on days 1 and 8. A pair of biopsies was taken from each cube using a 16-G biopsy instrument on both days 1 and 8 (Tru-Core II, Argon Medical, TX, USA), and core quality assessed visually.

#### Phantom design and construction

**Fig. 2:**
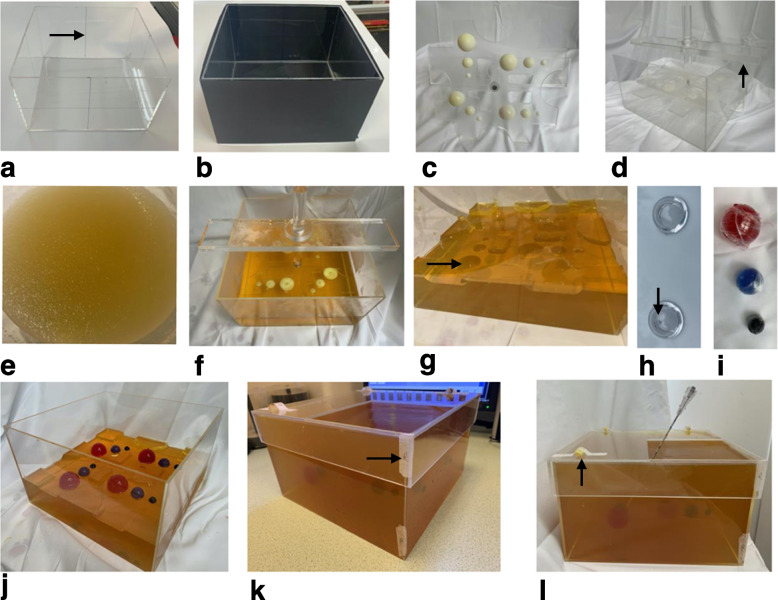
Phantom. Feature demonstration (**a–d**); construction steps (**j–l**). **a** Acrylic box with scribed lines (arrow) for scanner laser alignment. **b** Black blind to prevent observation during biopsy. **c** Plate with hemispheres for well impressions. **d** Plate in position, with flanges (arrow) to keep stamp 5 cm from base. **e** 1:10 gelatin:water, heated to 50 °C in a water bath. **f** Stamp in position, making wells in the half-filled gelatin as the gelatin cools. **g** Stamp removed, leaving wells (arrow) in the set gelatin in which the targets rest. **h** Two hemispherical domes form each sphere, with a 2-mm hole (arrow) in the apex of one of the domes which accommodates an 18-G needle for injection filling of target material. **i** Formed target spheres, filled with coloured gelatin/agar mix. Red, blue, and black food colouring for 3-, 2-, and 1-cm targets, respectively. **j** Spheres resting in wells. **k** Complete phantom, with gelatin filled to the top and surface stickers on the side of the phantom for orientation (arrow). **l** Phantom with biopsy needle *in situ*, with surface markers for confirmation of orientation (arrow)

A full description of phantom design, construction, and costings are provided in the Additional file 1. In brief, 1:10 gelatin was chosen as the background material, and equal parts 1:10-gelatin and 6:100-agar as the target material since these workable materials provided adequate cores, approximately matched HU (both around 25), and different *T*_2_ relaxation times (approximately 500 ms *versus* 200 ms respectively) to generate MRI contrast. A total of four identical biopsy phantoms were constructed using acrylic boxes (25 × 25 × 15 cm^3^) filled with background (non-target) dyed gelatin, with four sets of three spherical targets composed of mixed gelatin/agar (diameters 1, 2, and 3 cm) at 10-cm depth (12 targets in total), dyed with different food colourings to distinguish targets from background when assessing biopsy cores. A custom-made stamp was used to create wells as the background gelatin set, on which the targets were fixed at reproducible positions. A square aperture (13.5 × 13.5 cm^2^) in the lid mandated in-plane biopsy (Y displacement only, targets 1–3); single oblique (XY for targets 4–6 and ZY for 7–9), and double oblique (XZY for targets 10–12) approaches (Fig. 2). Two of the phantoms (A and B) were constructed one week apart for reproducibility assessment and technique development, and two further phantoms (C and D) were constructed for multioperator reliability assessment.

#### Phantom validation

Phantoms were imaged on the same MRI scanner as the trays using a three-dimensional T2-weighted TSE acquisition (axial, 104 slices, slice thickness 2.5 mm, echo time 140 ms, repetition time 1,500 ms, 1.4 signal averages, in-plane reconstructed pixel size 1.3 × 1.3 mm^2^, acquisition time 6 min 14 s). Target visibility was assessed visually while target position reproducibility was assessed qualitatively using image fusion (Horos, horosproject.org) and quantitatively using a Dice similarity coefficient (DSC) (MATLAB 2021a, Mathworks, Natick, MA) for phantoms A and B. All acquisitions were carried out the day after phantom construction.

### Robotic biopsy

A commercially available interventional CT robot (MAXIO™, Perfint Healthcare, Chennai, India), licensed for interventional procedures in the chest, abdomen, and pelvis was used to perform fusion biopsies [12]. The integrated planning workstation allows up to six needle trajectories to be planned using multiplanar reformats, and has work-in-progress MRI fusion software (off label use). Each planned trajectory is translated to object space by an electromechanical arm (stereotaxy), which has grippers that hold a needle guide, through which biopsy needles are manually placed. Rigid MRI/CT fusion was performed using the planning workstation by defining between 3 and 5 corresponding points-of-interest using fixed structures on both the MRI and planning CT images, namely the corners of the acrylic box.

#### Feasibility and pipeline

**Fig. 3:**
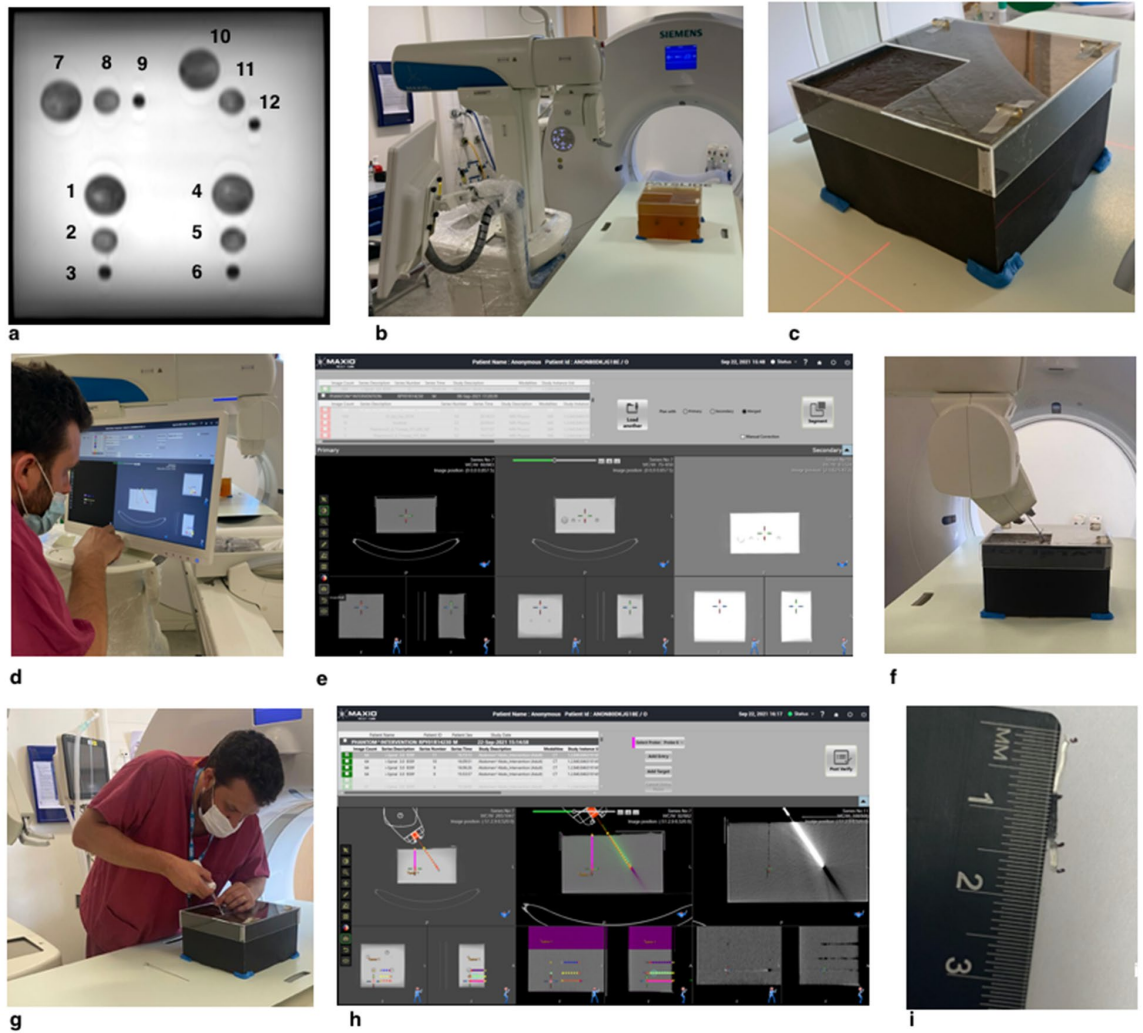
**a** Coronal/birds-eye on T2-weighted turbo spin-echo images with numbered targets. **b** Phantom on the CT table next to the robot. **c** Aligned phantom, fixed with adhesive putty. **d** MRI/CT-fusion and needle path planning using robot workstation. **e** Workstation screenshot showing CT (left), fused MR/CT (middle), and MRI (right) reference points. **f** Robot and inserted co-axial needle. **g** Radiologist performing a biopsy. **h** Robot workstation showing planned (left), actual (right), and fused (planned and actual, middle) needle paths. **i** Biopsy core with target (black) and non-target surround (very pale yellow); core length and target length are being measured. *CT* Computed tomography, *MRI* Magnetic resonance imaging

One of the authors (E.J. 4-year experience in interventional radiology, including 3 months of clinical robotic intervention) developed a technical pipeline (Fig. 3) by taking biopsies from the targets in phantom A, with steps fully described in Additional file 1.

#### Multioperator reliability assessment

Four operators each attempted biopsy of all targets twice (back-to-back), 5−7 days after manufacture. Two operators biopsied phantom C and two operators biopsied phantom D using a 16-G × 20-cm biopsy needle (Tru-Core II, Argon Medical, TX, USA) which has a maximum core length of 19 mm. Operators had different levels of experience: E.J. (again, following pipeline development), N.F. (19-year experience with biopsy procedures including 3 months of clinical robotic intervention), J.L. (in-training, 2-year experience with biopsy procedures, including two months of clinical robotic intervention), J.W. (MRI physicist with no prior experience of conducting biopsy procedures but 11-year experience in medical imaging). Operators were blind to each other’s biopsies, apart from E.J. who attended all procedures to explain some aspects of software functionality, without physical input.

#### Freehand biopsy

After the robotic biopsies were carried out, a 16-G co-axial needle was positioned into the phantom in multiple (> 100) non-target regions of phantom D, to introduce non-target gas locules and make the location of targets uncertain. The quickest robotic operator then attempted freehand biopsy on day 7 after manufacture under CT guidance using low-dose sequential acquisitions and cognitive fusion, where measurements were translated from MRI to CT to estimate target position. As many targets as possible were biopsied in a 1-h time limit, starting with the 3-cm targets.

#### Biopsy core analysis

Cores were measured immediately after each biopsy (to minimise desiccation) by an independent operator (J.B.), in consensus with an operator not performing the biopsy procedure, to minimise bias. Each core was straightened (if necessary) on a piece of white card, and core length (CL)–the total length of the biopsy core (target + background material) and target length (TL)–(target material length only) marked alongside the core using an ultrafine (0.1 mm) black tip pen (adapted from [13]), and measured using a ruler to the nearest 0.5 mm.

### Statistical analysis

Statistical analysis was carried out using GraphPad Prism (version 9, San Diego, CA) and MATLAB (2021a, Mathworks, Natick, MA). A *p* value of < 0.05 was used to indicate statistical significance.

Data were checked for normality using the Shapiro–Wilk test, and column statistics calculated. Repeatability of T1_,_ T2, and HU in trays and phantoms was assessed using Bland–Altman analysis [14] and time taken to perform the first *versus* second phantom biopsies compared using paired *t* tests.

**Fig. 4:**
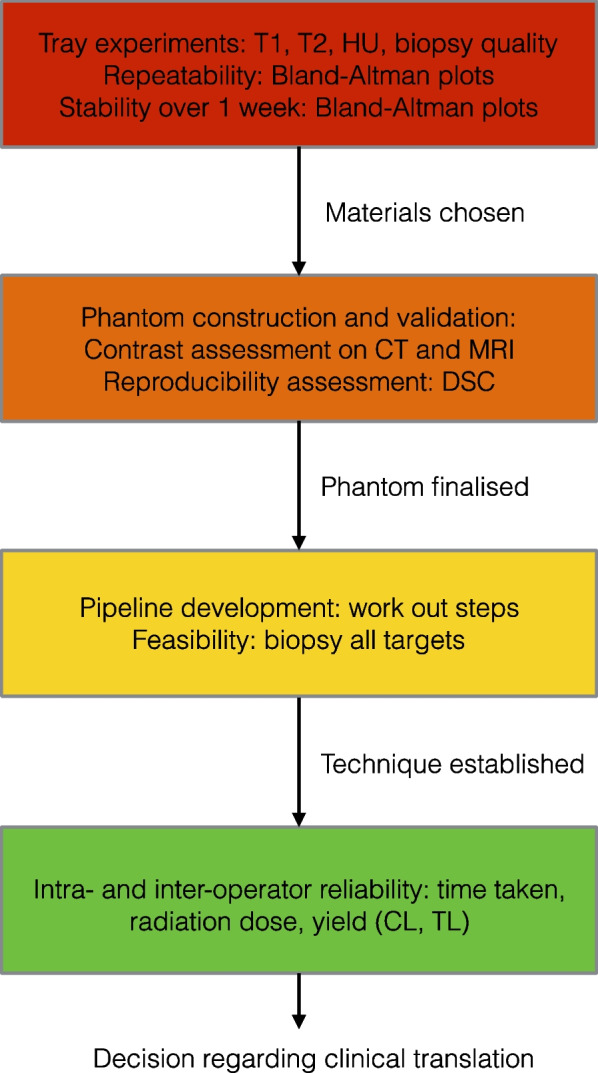
Flow diagram of study methods. *CL* Core length, *DSC* Dice similarity coefficient, *HU* Hounsfield units, *TL* Target length

A flow diagram of study methods is shown in Fig. 4.

## Results

### Tray experiments

**Fig. 5:**
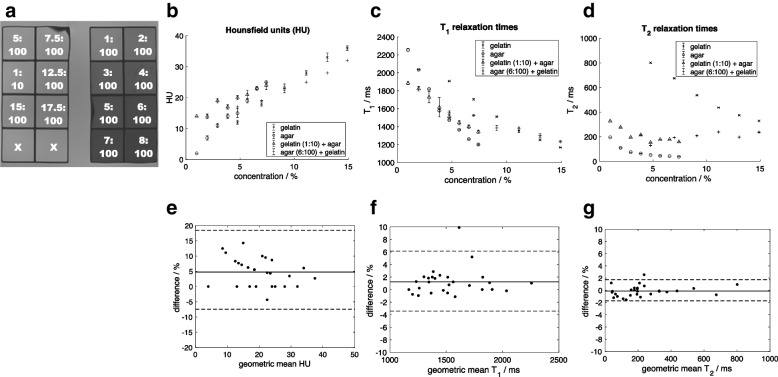
**a** Coronal T2-weighted (echo time 50 ms) magnetic resonance images of pure gelatin (left) and agar (right), trays 1 and 2, showing that gelatin qualitatively returns high signal at all concentrations, whereas agar returns lower signal as concentration increases (the bottom two wells were filled with doped water). Attenuation values (HU) (**b**) T1 (**c**), and T2 (**d**) at different gelatin/agar concentrations. These graphs confirm relatively small differences in HU as the concentration of both substances increases and show that the T2 relaxation time is long in all gelatin samples and short in agar samples. Bland–Altman plots comparing differences between the two test–retest measurements for HU (**e**) T1 (**f**), and T2 (**g**). Solid lines represent the mean difference, and dashed lines the 95% limits of agreement

T1, T_2_, and HU demonstrated strong dependence on gelatin and agar concentration and good repeatability with lower with upper 95% limits of agreement of -2.8 and 5.5%, -1.7% and 1.7%, and -3.8% and 14.1% respectively (Fig. 5).

There was a tendency for greater core integrity with higher substance concentration at the expense of higher cost and difficulty dissolving substances. Biopsy core quality did not differ between days 1 and 8, confirming material stability.

### Phantom validation

**Fig. 6:**
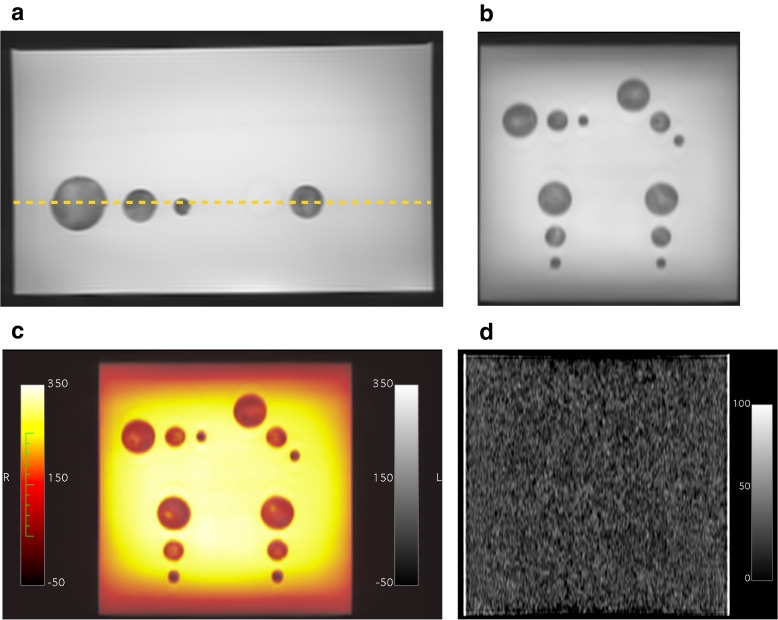
Biopsy phantom. **a** Axial/side view T2-weighted magnetic resonance image, with dashed yellow line showing location of coronal reformats. **b** Coronal/birds’ eye view reformat of T2-weighted images. **c** Fused magnetic resonance images of two separate biopsy phantoms showing the same position of targets. **d** Computed tomography appearances using a narrow soft tissue (brain) window to confirm targets are invisible

There was excellent contrast between targets and background material on MRI, without discernible CT contrast (Fig. 6). Target size and position highly reproducible between phantoms A and B, with a DSC of 0.92 (0.93 for scanning the same phantom).

### Robotic biopsy

Technique feasibility was initially demonstrated by successfully taking 12 biopsies in 89 min with 100% hit rate (12/12), including scanning after each needle insertion, which showed from 0.0 to 1.0 mm of tip deviation.

At multioperator assessment, visible target material > 1 mm was achieved in all attempted biopsies (hit rate 100%, 96/96). Median CL was 15.6 mm (interquartile range [IQR] 14.6−17.0) across all targets. Median TL was 15.0 mm (IQR 14.0–17.3) for 3-cm targets, 15.0 mm (IQR 10.0–6.0) for 2-cm targets, and 7.8 mm (IQR 6.6–9.0) for 1-cm targets. The maximum CL, defined by sample notch size, was 19 mm. The second set of biopsies was significantly quicker than the first, (61 min 0 s ± 27 min 42 s [mean ± standard deviation] *versus* 49 min 30 s ± 20 min 30 s, *p* = 0.049), with the longest at 101 min (MRI physicist) and the quickest time at 32 min (attending radiologist) for all 12 biopsies, from start to finish (scanning, image fusion, and biopsy). Each set of biopsies was performed using a single volumetric acquisition, mean dose-length product of 145 mGy*cm (range 140–150), with a single needle positioning for each biopsy (*i.e.*, no readjustments).

Using freehand guidance using cognitive image fusion, 3 targets (1, 5, and 12) were successfully biopsied, and 1 was missed entirely (target 9), with total dose-length product of 675 mGy*cm in a 1-h time slot, which is 8 times longer per target, and 18 times higher radiation dose per target than the fastest robotic operator.

## Discussion

In this study, we sought to develop and rigorously validate a technique for MRI-guided biopsy in the CT scanner using image fusion and stereotactic targeting to better decide whether it is suitable for translation to clinical studies.

We first developed and validated a unique purpose-built, low-cost, non-toxic, MRI/CT fusion biopsy phantom, which had 12 dyed targets of varying difficulty levels that could be seen on MRI but not on CT, can be biopsied, and core adequacy assessed for presence of dyed target material. The similar DSC for two separate phantoms compared with two MRI scans of the same phantom (0.92 and 0.93) means that our well-controlled construction process yielded highly reproducible phantoms, which is crucial to maintain consistent difficulty and compare true operator performance.

Intraoperator and interoperator reliability can be readily assessed using phantoms, since they can be biopsied more than once, which allows risk to be minimised through training and development or abandonment of unsuccessful techniques. After showing initial feasibility, we tested reliability in a range of operators with different experience levels, including an MRI physicist without prior experience of performing biopsies. All operators biopsied targets with 100% (96/96) hit-rate including highly complex 20°–40° double oblique angulations in small targets, using a single volumetric acquisition and a clinically acceptable duration of around 30 min in the fastest operator, demonstrating that our technique is reliable and therefore suitable for clinical translation. The ability to select and execute complex oblique needle trajectories using multiplanar reformats is a key advantage of our robotic technique, which provides more available needle path options, and allows vital structures to be avoided.

The diagnostic yield of conventional CT-guided biopsy for lesions less than 1 cm in diameter is limited [15–18] without the added complexity of out-of-plane paths and image fusion. Indeed, our 0.0−1.0-mm tip deviation is highly accurate and in line with the results reported by Scharll et al. [19], who quoted needle placement accuracy of 1.5 ± 0.87 mm (mean ± standard deviation) in a structurally simple gelatin phantom with embedded aluminium targets, without MRI/CT fusion. Our studies are more accurate than another group who used the same robot without image fusion and a commercially available hydrogel polymer phantom, and reported a tip deviation of 6.5 ± 2.5 mm [12], which could be due to differences in phantom materials (hydrogel polymer gelatin), analytical methods, and a greater internal complexity of their phantom, with resemblance to anatomical structures.

Limitations of the phantom stem from its differences with patients including lack of movement, object simplicity making fusion straightforward, poor anatomical resemblance, and degradation of the phantom with use. Whilst needle path tracks were sometimes faintly visible on CT, they were not used to guide biopsy in our experiments. Their minimal impact is evidenced by the fact cores were successfully obtained in all targets on first attempt using the robotic technique.

Further work regarding phantom development could include reproduction at other centres, use for in-gantry biopsy and three-dimensional printing patient-specific phantoms [20]. Clinical translation should include the addition of a vacuum immobilisation mattress to reduce external motion [21]. We also advise taking a stepwise approach, where relatively fixed tumours are first investigated (*e.g.*, MRI-guided bone biopsy [22, 23], followed by more mobile tumours using internal motion correction techniques as has been applied in robotic lung biopsy without image fusion using breath hold monitoring [24]. For example, whereas rigid registration (as used in our study) might be more suitable for non-deformable structures with fixed internal points (*e.g.*, bone), deformable structures may benefit from more complex deformable registration algorithms [25].

If successful, MRI/CT fusion in biopsy procedures could allow the most deterministic intratumoural components to be targeted for more accurate classification and better management decisions. Tumour targeting in ablation procedures, combined with apnoea or high-frequency jet ventilation [26] would be improved for index tumours that are occult on CT, but not on MRI [27]. Consequently, there is an unmet clinical need for the development and validation of fusion software in combination with stereotactic interventional devices.

In conclusion, a reproducible biopsy phantom was successfully developed, validated, and used to rigorously test a robotic technique for MRI/CT-fusion biopsy. The technique was shown as highly accurate, reliable, and suitable for clinical translation.

## Supplementary Information

**Additional file 1: Supplemental Table 1.** Phantom costings. **Supplemental Figure 1.** Targets accessible via in plane approach (red arrow), single oblique (green arrow) and double oblique approach (dotted blue arrow) as mandated by the box lid, and its opening. Note the ‘F’ markings on the side of the box and lid, denoting the ‘feet’ for orientation purposes (black arrowheads). **Supplemental Figure 2.** Design features of the phantom. A: schematic birds eye view of the phantom stamp. B: CAD model of the under surface of the stamp. White asterisks demonstrate defects which reduce surface area and thus interfacial tension between the stamp and gelatin, facilitating removal. C. CAD model of hemispheres (1, 2 and 3cm) + locating pegs. **Supplemental Figure 3.** The stamp hanging down onto gelatin as it sets, to create well imprints in the set gelatin.

## Data Availability

Available from the corresponding author on request.

## Abbreviations

CL: Core length
CT: Computed tomography
DSC: Dice similarity coefficient
HU: Hounsfield units
IQR: Interquartile range
MRI: Magnetic resonance imaging
TL: Target length
TSE: Turbo spin-echo
