# Supplementary material for: Developing and testing a robotic MRI/CT fusion biopsy technique using a purpose-built interventional phantom
Source: Eur Radiol Exp. 2022 Nov 22;6:55. doi: 10.1186/s41747-022-00308-7 (PMC9679095; doi:10.1186/s41747-022-00308-7)
Supplement: Supplementary file 1 — Additional file 1: Supplemental Table 1. Phantom costings. Supplemental Figure 1. Targets accessible via in plane approach (red arrow), single oblique (green arrow) and double oblique approach (dotted blue arrow) as mandated by the box lid, and its opening. Note the ‘F’ markings on the side of the box and lid, denoting the ‘feet’ for orientation purposes (black arrowheads). Supplemental Figure 2. Design features of the phantom. A: schematic birds eye view of the phantom stamp. B: CAD model of the under surface of the stamp. White asterisks demonstrate defects which reduce surface area and thus interfacial tension between the stamp and gelatin, facilitating removal. C. CAD model of hemispheres (1, 2 and 3cm) + locating pegs. Supplemental Figure 3. The stamp hanging down onto gelatin as it sets, to create well imprints in the set gelatin. [file 41747_2022_308_MOESM1_ESM.pdf]

ELECTRONIC SUPPLEMENTARY MATERIAL

Developing and testing a robotic MRI/CT fusion biopsy technique using a purpose-built interventional phantom

The leading commercially available interventional phantom is designed for separate multimodality use such that targets are visible on CT, US, and MRI, making it unsuitable as a fusion phantom. We therefore designed a phantom that has: i) targets visible on MRI but occult on CT ii) can be physically biopsied for assessment of cores iii) different target difficulty levels but accessible with standard biopsy needles iv) stability i.e., doesn't change properties or perish over a period of use v) low cost vi) reproducibility for consistency and scalability.

We provide a full description of how the phantom was designed and constructed, as to increase reproducibility for other investigators wishing to conduct similar work. Please contact the corresponding author if you would like to purchase a stamp from our workshop (not for commercial use).

1. Background

- The phantom casing is a 25cm (width) x 25cm (length) x 15cm (height) acrylic box with a lid, cut down in height by a laser cutter or circular saw from a 25cm cube acrylic cube (removing 10cm from the top). Acrylic cubes with lids are readily purchasable online.

- We decided the phantom should be refillable using cheap materials, such that biopsies can be taken without degradation being a concern, unlike commercially available phantoms.

- We wanted a range of 'difficulty levels' – round (tumor-like) targets of 1, 2 and 3cm to correspond with lesion sizes that we might expect to biopsy clinically, accessible using a mix of in plane, single and double oblique approaches of around 20 – 40 degrees

angulation. Dimensions were calculated to ensure all targets would be reachable by a 14.8cm co-axial needle from a 13.5cm square opening in one of the corners of the lid (i.e., larger than a quadrant), to mandate in plane, oblique and double oblique approaches (figure 1). This opening was cut with a laser cutter.

- To keep the position of the spheres consistent when phantoms are refilled, we made a stamp with embedded hemispheres the size of the targets attached to a plate, which could be placed upon liquid gelatin, and then removed when set, leaving wells in which the targets would rest (figure 2).

2. Design and construction of reusable materials

- Plate and hemisphere geometry was calculated and drawn using a computer aid design package (Autodesk Inventor 2019, CA, USA). The CAD model was then used to laser cut the plate to which the hemispheres are fixed, and 3D print the hemispheres using a 3D printer (Objet Eden 350V using MED610, Stratasys, IL) which were fixed to the main plate using locating pegs (figure 2).
- Markings were scribed to mark the center points of each side of the acrylic box to align with the scanner lasers.
- A blind was made from black card which surrounded the acrylic casing to prevent observation during biopsy procedures.
- The plate was attached to a hanging rod, which hangs from above and rests on flanges on the sides of the box, a consistent 5cm from the base (figure 3).
- Markers were placed on the sides of the phantom (H - head, F - feet, L - left and R - right for orientation purposes. When making the phantom, it is important to ensure the stamp is orientated the correct way (figure 1).

The following items must be purchased to fill the phantom:

- Gelatin powder (1kg per phantom), 250 bloom (high gelling strength).
- Agar powder (500g will make 55 phantoms at 9g per phantom).
- Food coloring (yellow, 30mls per phantom, red, blue and black in smaller quantities <5mls per phantom).
- Large mixing bowls for the gelatin.
- A thermometer, 0 – 100 degrees.
- Precise scientific weighing scales for weighing agar (e.g., 0.01g to 500g) (kitchen scales suffice for gelatin).
- Blunt 18G needles and 10ml syringes.
- Plastic hemispherical domes, 1, 2 and 3cm internal diameter (eight to twelve of each), whereby half should be prepared by drilling a 2mm hole at the apex to accommodate a needle and syringe for filling with gelatin/agar mix.
- Plastic wrap (‘cling film’).

3. Recipe for (re)filling the phantom (background and targets)

The following instructions should be followed each time a biopsy session is required, since the phantom will degrade with time and biopsy procedures. Steps can be carried out in a domestic kitchen (and were, due to the closing of our laboratories during the COVID-19 pandemic).

Making the gelatin base:

- Weigh 1kg of gelatin and scatter on the surface of 10 liters of water. This process can be done in two bowls of 500g gelatin and 5 liters of water in each bowl. The mix is then left to rest, for an hour, until translucent in a process called ‘blooming’.
- The 1:10 gelatin mixture is then heated in a water bath until transparent and approximately 50°C (as measured with a scientific thermometer). Do not boil the gelatin.

- 500mls gelatin is removed and kept for manufacture of the targets.
- 3.3mls/liter of food coloring is added to the rest of the gelatin for the background substance – we used yellow food coloring.
- Mark the level of the hanging stamp in the phantom, and remove the stamp
- Pour the liquid yellow gelatin into the box up to the level of the stamp (just over 3 liters)
- Reposition the stamp and top up with more liquid gelatin as required such that the stamp is just covered.
- Transfer the whole system to the refrigerator until fully set and very firm. This process will take at least two hours.
- Whilst the gelatin is setting, the targets can be made (see below).
- Once set, remove the stamp, and inspect the wells left by the stamp. The wells should not lose their shape at all. If they do, the gelatin is not set enough.

Making the targets:

- Wrap all the pairs of hemispherical domes (one with a hole, one without) in cling film to form spheres.
- Weigh 9g agar, and add to 150mls water
- Microwave the mixture in a cup until it is boiling, stirring intermittently though interrupted microwave cycles to mix the 6:100 solution evenly.
- Once fully dissolved, mix the agar with an equal quantity of the uncolored gelatin you saved from earlier, and stir well to mix the substances.
- Separate into three utensils and dye with a few drops of food coloring in each (we used red, blue, and black), until the mixture takes on the color of the food coloring. Do not add too much food coloring, or the density of the balls will change, and the targets will become visible. We used 3 different colors to confirm the correct targets were being biopsied.
- Draw up the dyed target mixture with a syringe, and then attach the blunt needle.

- Inject the spheres with the gelatin/agar mix, and place into a freezer until fully set.
- Carefully remove the targets from the hemispheres. The 1cm spheres in particular can be damaged in this process so it is advisable to make more than four of this size e.g., six of each.
- Once the targets have been made, trickle some yellow liquid gelatin into each of the wells using a syringe, and place the spheres onto each of the relevant wells, until the spheres are stuck into place. This process should be relatively quick (a few minutes) since the gelatin base is cool from refrigeration.
- Fill the rest of the phantom up with the rest of the background gelatin (just over 6 liters), taking extreme care in this process since the targets are very delicate. Do not pour the additional gelatin onto the targets directly or knock the phantom casing, otherwise there is a high chance of targets losing their integrity, ruining the phantom. This process of pouring can be carried out in the refrigerator to minimize knocks during transfer. It is also possible to carry out this process using a two-step approach, pouring background substance to cover the targets, letting it set, and then pouring the rest of the gelatin to fill the rest of the phantom.

Supplemental table 1. Phantom costings

Each phantom cost £175 up-front, and £17 (gelatin only) for each refill.

Item	Cost per item (£)	Quantity	Total cost (£)
Acrylic box with lid	42.98	1	42.98
Custom made stamp	68.00	1	68.00
Gelatin 1kg	16.99	1	16.99
Agar 500g	4.99	1	4.99
3cm hemisphere	1.58	8	12.64
2cm hemisphere	0.92	10	9.2
1cm hemisphere	0.67	12	8.04
Black card	7.99	1	7.99
Food coloring	1.30	3	3.90
Grand total	145.42		174.73

5. Steps for performing MRI/CT fusion biopsy

- Switch on and dock the device
- Transfer the T_2 weighted DICOM MRI images to the robot using a USB device.
- Orientate the phantom the same way as MRI (guided by the aperture and the surface stickers).
- Align the scribed lines on the external faces of the phantoms to the isocentre of the CT scanner, in the same fashion as for MRI.
- Grip the phantom to the CT couch using adhesive putty to minimize movement.
- Scan the phantom using the previously described CT acquisition.
- Send DICOM CT images to the device.
- Use the planning workstation to fuse the MRI and CT images using a point-based registration, choosing 3 – 5 suitable landmarks (the edge of the box).
- Check fusion accuracy using a sliding bar on the software which fades between MRI and CT images. The fusion process can be repeated at this point if judged as inaccurate.
- Once fusion is deemed satisfactory, plan the first six needle paths, in ascending level of difficulty i.e., targets 1 – 6.
- Position the device in the first biopsy position and place a co-axial needle (15G x 14.8cm Tru-Core II, Argon Medical, TX, USA).
- Take a biopsy core using a 16G x 20cm biopsy needle (Tru-Core II, Argon Medical, TX, USA). N.B. this device has a maximum core length of 19mm.
- Analyze core adequacy for presence of colored target material. If target material is achieved, remove the co-axial needle.
- If desirable e.g., no target material is seen in the biopsy core, the phantom can be scanned again and the planned vs. actual tip/path deviation can be measured using planning CT / post needle insertion CT image fusion on the MAXIO workstation. This was performed following each co-axial needle insertion during pipeline development.

- Reposition the device for the second biopsy and repeat the process until the 6th target is biopsied.
- Plan the needle paths for targets 7 – 12, which requires repeat fusion.

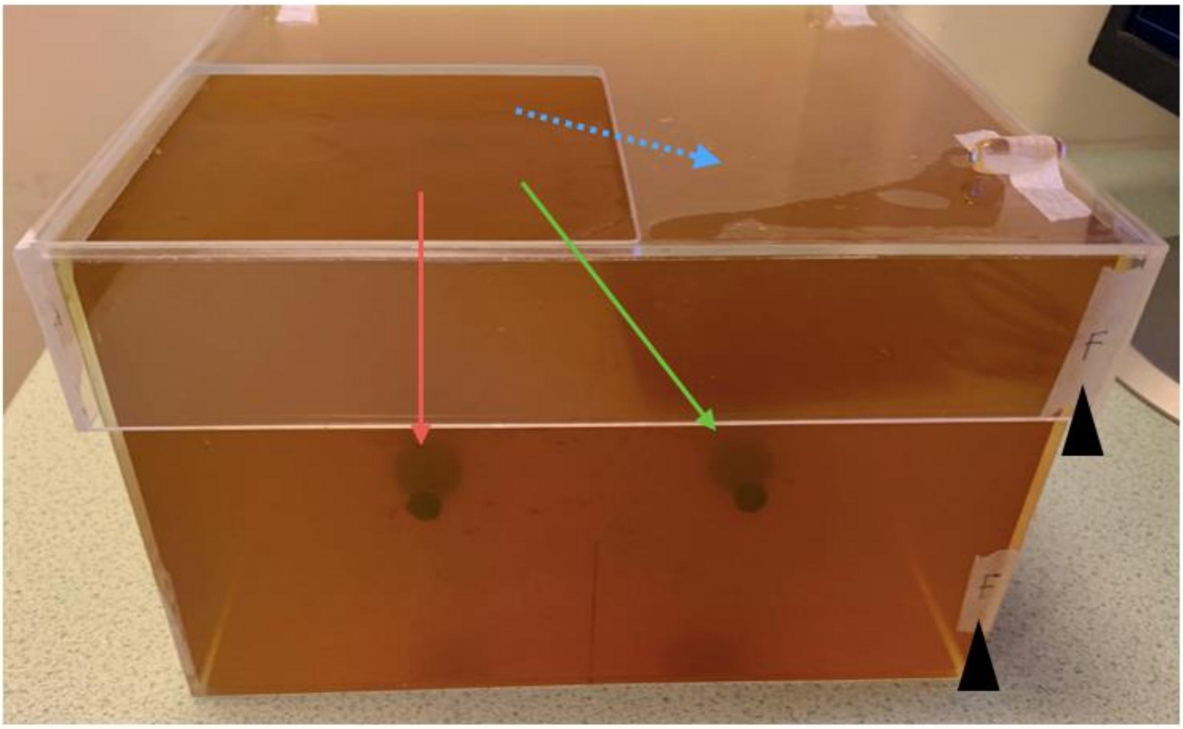

Supplemental Figure 1. Targets accessible via in plane approach (red arrow), single oblique (green arrow) and double oblique approach (dotted blue arrow) as mandated by the box lid, and its opening. Note the ‘F’ markings on the side of the box and lid, denoting the ‘feet’ for orientation purposes (black arrowheads).

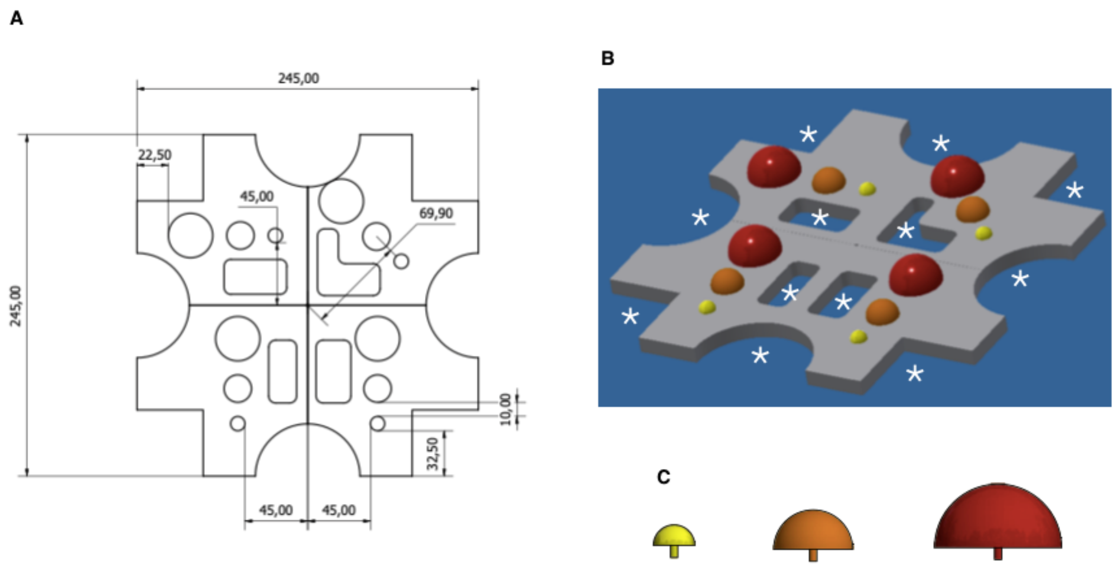

Supplemental Figure 2. Design features of the phantom. A: schematic birds eye view of the phantom stamp. B: CAD model of the under surface of the stamp. White asterisks demonstrate defects which reduce surface area and thus interfacial tension between the stamp and gelatin, facilitating removal. C. CAD model of hemispheres (1, 2 and 3cm) + locating pegs.

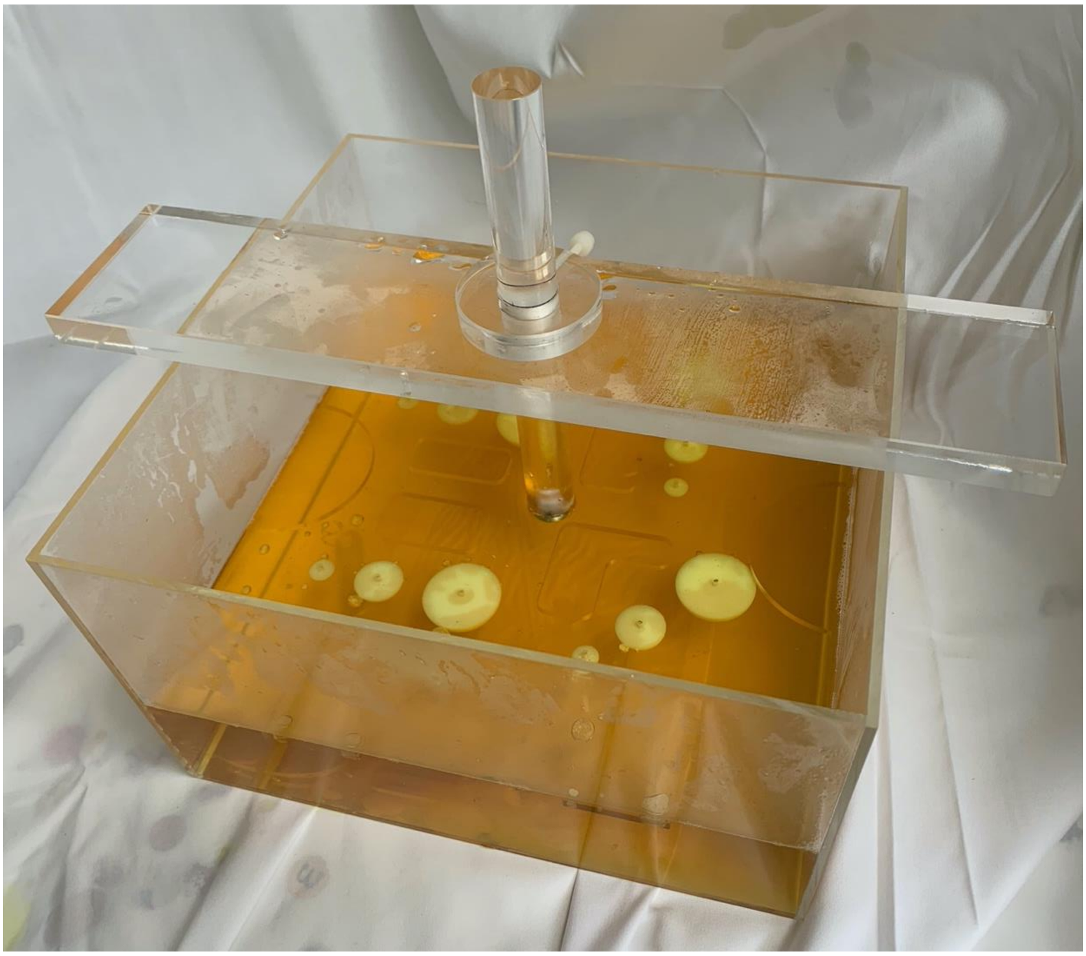

Supplemental Figure 3. The stamp hanging down onto gelatin as it sets, to create well imprints in the set gelatin.
